# The effect of electrical stimulation in critical patients: a meta-analysis of randomized controlled trials

**DOI:** 10.3389/fneur.2024.1403594

**Published:** 2024-07-31

**Authors:** Lili Li, Fei Li, Xinyin Zhang, Yuying Song, Shuyan Li, Huiping Yao

**Affiliations:** Emergency and Critical Care Center, Intensive Care Unit, Zhejiang Provincial People’s Hospital, Affiliated People’s Hospital, Hangzhou Medical College, Hangzhou, Zhejiang, China

**Keywords:** electrical stimulation, ICU, meta-analysis, physical therapy, randomized controlled trial

## Abstract

**Objectives:**

While electrical stimulation has been demonstrated to improve medical research council (MRC) scores in critically ill patients, its effectiveness remains a subject of debate. This meta-analysis aimed to discuss recent insights into the effectiveness of electrical stimulation in improving muscle strength and its effects on different clinical outcomes in critically ill adults.

**Methods:**

A comprehensive search of major electronic databases, including PubMed, Cochrane Library, and Embase, was conducted from inception to June 15, 2024, to identify randomized controlled trials (RCTs) that evaluated the effects of electrical stimulation in critically ill patients. The analysis focused on comparing electrical stimulation to standard care, sham interventions, or placebo. Outcomes of interest included MRC scores, duration of mechanical ventilation (MV), mortality rate, and intensive care unit (ICU) and hospital length of stay (LOS).

**Results:**

A total of 23 RCTs, including 1798 patients, met the inclusion criteria. The findings demonstrated a significant benefit of electrical stimulation over usual care in enhancing global muscle strength, as measured by MRC scores (MD =3.62, 95% CI 0.94 to 6.30, *p* = 0.0008, I^2^ = 87%). While subgroup analysis of electrical muscle stimulation (EMS) demonstrated no significant effect on ICU LOS, sensitivity analysis indicated a potential reduction in ICU LOS for both EMS (MD = −11.0, 95% CI −21.12 to −0.88, *p* = 0.03) and electrical stimulation overall (MD = −1.02, 95% CI −1.96 to −0.08, *p* = 0.03) compared to the control group. In addition, sensitivity analysis suggested that both electrical stimulation (MD = −2.38, 95% CI −3.81 to −0.94, *p* = 0.001) and neuromuscular electrical stimulation (NMES) specifically (MD = −2.36, 95% CI −3.85 to −0.88, *p* = 0.002) may contribute to a decrease in hospital LOS. No statistically significant differences were observed in mortality or duration of MV.

**Conclusion:**

Electrical stimulation appears to be an effective intervention for improving MRC scores in critically ill patients. However, further research is warranted to explain the potential effects of electrical stimulation on hospital LOS and ICU LOS.

**Systematic review registration:**

https://www.crd.york.ac.uk/PROSPERO/#recordDetails.

## Introduction

ICU-acquired weakness (ICU-AW), a debilitating condition characterized by muscular weakness arising from a confluence of risk factors in intensive care unit (ICU) stays, is a prevalent concern. Studies indicate that the incidence of ICU-AW in critically ill patients can reach up to 70% (1). This condition has significant implications for patient outcomes, correlating with extended durations of mechanical ventilation (MV), protracted ICU and hospital lengths of stay (LOS), increased hospital mortality rates, and the persistence of debilitating weakness (2, 3). Proactive interventions for ICU patients are considered crucial in reducing the development of ICU-AW (4). Early implementation of active rehabilitation methods has demonstrated effectiveness in enhancing muscle strength and mobility, simultaneously reducing hospital LOS and mortality rates among ICU patients (5, 6). However, the feasibility and extent of early functional training can be limited by the severity of a patient’s medical condition and their capacity for active participation. Electrical stimulation presents a non-invasive and safe alternative, particularly valuable for patients in the early stages of their ICU stay, especially those who are unconscious or necessitate sedation (7). Research suggests that electrical stimulation confers a therapeutic advantage in managing ICU-AW, leading to increased muscle strength, shortened MV durations, and reduced ICU LOS (8, 9); whereas, a subset of studies has reported no significant improvements in muscle strength attributable to electrical stimulation in ICU-AW (2, 10, 11). Therefore, despite the widespread clinical adoption of electrical stimulation, its effectiveness for ICU patients continues to be a subject of debate.

A prior systematic review (2), which considered studies published through 2020, comprised six randomized controlled trials (RCTs) identified through a search concluded in November 2018. Since then, new analyses in this area have been conducted. For instance, Zayed et al. (2) studied adult patients admitted to the ICU for medical or surgical reasons, irrespective of their need for MV, and found that integrating electrical stimulation into standard care did not yield significant differences in muscle strength, ICU mortality, MV duration, or ICU LOS compared to standard care alone for critically ill patients; whereas, Baron et al. (12) and Chen et al. (3) demonstrated that electrical stimulation could potentially shorten ICU LOS.

The purpose of this review was to present recent findings on the effectiveness of electrical stimulation for enhancing muscle strength and its impact on various clinical outcomes in critically ill adults.

## Methods

The research adhered rigorously to the guidelines set forth by the Preferred Reporting Items for Systematic Reviews and Meta-Analyses (PRISMA) statements (13). Furthermore, it was officially registered with PROSPERO on September 12, 2022, and was assigned the registration number CRD42022350794. This study protocol was registered after the first literature search.

### Search strategy

A systematic search of literature and electronic databases such as PubMed, Cochrane Central Register of Controlled Trials, and Embase was conducted from their inception until June 15, 2024. The initial literature search was conducted on July 7, 2022. The strategy to develop search terms involved a blend of subject terms and freely used words. This includes terms like “electric stimulation therapy,” “intensive care units,” “critical illness,” and “ICU.” A detailed combination of free words and subject terms was utilized for retrieving literature, and the specifics of this search strategy can be found in Appendix 1.

### Inclusion and exclusion criteria

The inclusion criteria were: (1) Study type: RCT, not limited to allocation concealment and blinding method; (2) Study population: ICU mechanically ventilated patients aged ≥18 years; (3) Interventions: Research electrical stimulation or combined conventional therapy in the observation group; (4) Comparisons: Usual care measures or comfort treatment in the control group; (5) Outcome: The primary outcome was the Medical Research Council (MRC) scale score, while the secondary outcomes were the duration of MV, mortality, ICU LOS, and hospital LOS; and (6) Language: Only articles published in English.

The exclusion criteria were: (1) conference abstracts; (2) case studies or Meta-analyses; and (3) studies where data were missing or could not be converted.

### Literature screening and data extraction

A pair of reviewers separately perused through the scholarly records using EndNote 20.0, concurrently validated the compiled data, and sought judgment from a third-party researcher during disparities. The process of screening the literature implied a thorough examination of the title, abstract, and the complete text. In situations where critical information, necessary for the study was missing, the original authors of the papers were reached out to, either via email or call. Excel was employed for data organization included several components such as the authors’ names, year of publication, Acute Physiology and Chronic Health Evaluation II (APACHE II), sample size, age demographics, gender, intervention strategies, and final outcomes.

### Literature quality evaluation

Two reviewers conducted independent assessments of the risk of bias in the studies included in the review using the risk of bias assessment tool for RCTs as outlined in Cochrane Workbook 6.4, 2023 (14). In cases where there was a difference in their assessments, a third investigator was involved in discussions or arbitration to reach a consensus. The evaluation encompassed various aspects, including random sequence generation, allocation concealment, blinding of participants and investigators, blinding of outcome assessors, completeness of outcome data, selective reporting and other potential sources of bias. Each of these aspects was rated as “low risk of bias,” “unclear,” or “high risk of bias,” with a determination of “high risk of bias” made for each specific item where applicable.

### Statistical analysis

We derived risk ratios (RRs) for categorical data and evaluated mean differences (MDs) for ongoing data, pairing them with their respective 95% confidence intervals (CIs) under a random-effect model. We gauged heterogeneity across studies using metric likeτ2, χ^2^ (Cochrane Q), and I^2^ statistics. According to the Cochrane handbook, the I^2^ will be considered non-important (<30%), moderate (30–60%), and substantial (>60%) (14). Our findings were represented visually through forest plots. To evaluate the effect of individual studies on the overall results, we employed a sequential study removal method, iteratively excluding each study and recalculating the pooled effect size. For gauging publication bias, we used a funnel plot for Meta-analysis and employed Egger’s method for quantification, provided more than ten studies incorporated the results. We also executed subgroup evaluations focusing on the various electrical stimulation forms. Our statistical reviews were performed using the RevMan 5.4 software, establishing a statistical significance benchmark at a value below 0.05.

## Results

### Summary of included studies

**Figure 1:**
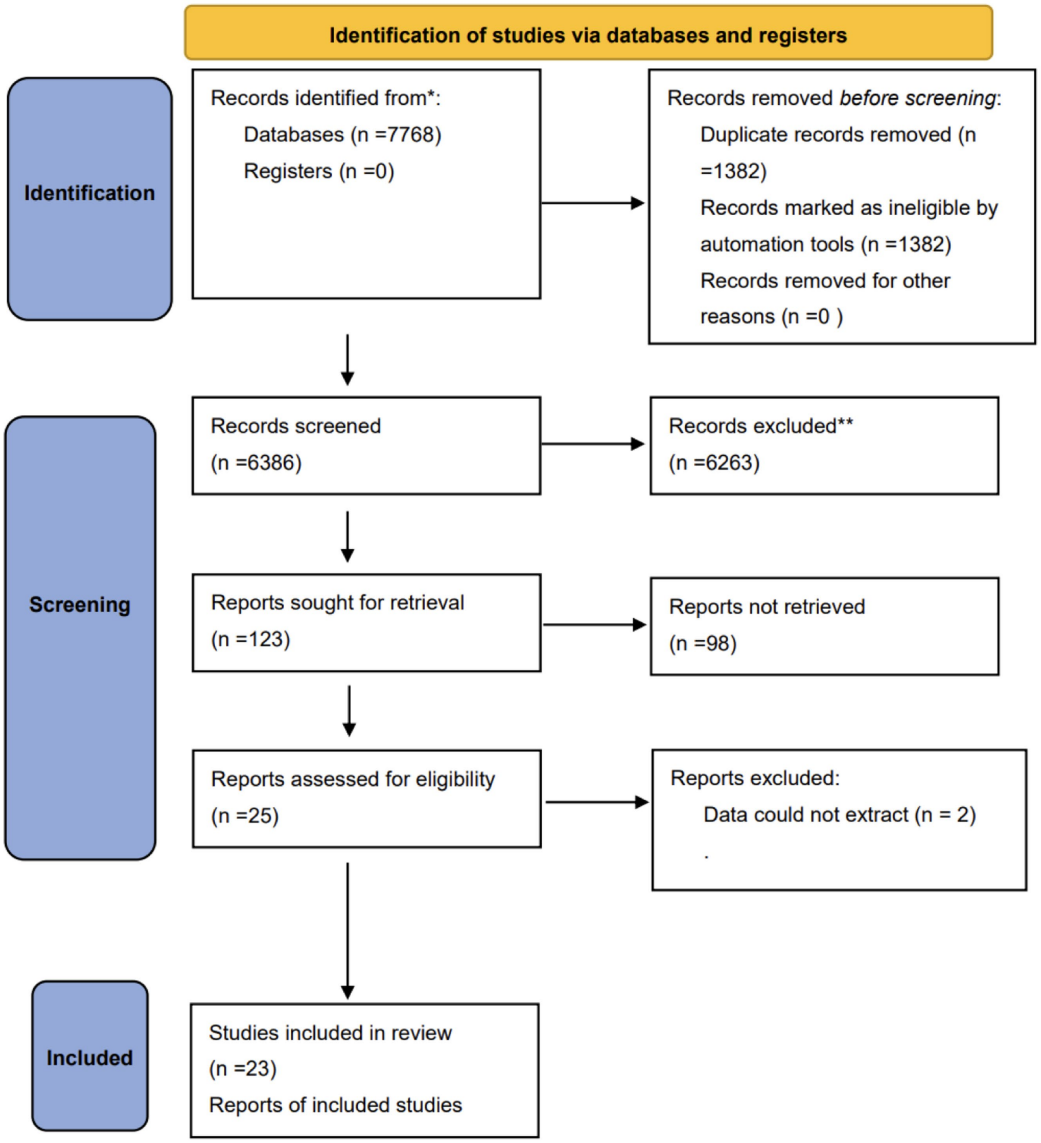
Flow diagram for literature search and study selection.

We identified a total of 7,768 studies related to our research. After checking for duplicates, screening titles, and browsing abstracts, we eliminated 7,745 studies that did not meet our inclusion criteria. The search process and study selection are illustrated in Figure 1. In the end, 23 randomized controlled trials (RCTs) (3, 12, 15–35) were included, covering a total of 1798 patients, of which 48.5% were females, conducted in 10 different countries and published between the years 2009 and 2023. The individual studies included a sample size range between 20 to 312 critical patients, and nearly 30.4% of the studies involved patients whose mean or median age was over 60 years.

The interventions in these trials were neuromuscular electrical stimulation (NMES) in 16 studies, electrical muscle stimulation (EMS) in five, and functional electrical stimulation (FES) in two. Notably, Abu-Khaber et al. (15) compared the effects of EMS with a placebo, Kho et al. (16) and Fischer et al. (17) compared it with a sham intervention, and Campos et al. (18) compared it with early mobilization. Table 1 provides additional details of the research and the clinical characteristics of the patients. This study employed a leave-one-out approach, removing the included studies one by one to observe changes in the results after the exclusion of specific studies, in order to examine the impact of any single study on the overall effect estimate. The results are shown in Table 2.

**Table 1:** Characteristics of included studies.

Study	Country	NMES group						Control group					Outcome
		APACHE II	Sample size	Age (year)	Female (%)	Intervention (NMES/EMS/FES)	Treatment in the experimental group	APACHE II	Sample size	Age (year)	Female (%)	Intervention	
Abu-Khaber, 2013 (15)	Egypt	24.5 ± 6.8	40	59.07 ± 5.32	24 (60%)	EMS	Frequency: daily, from the second day after admission Intensity:50 Hz, pulse width of 200 μs	26.1 ± 5.3	40	57.57 ± 6.80	27 (67.5%)	Placebo	Mortality Duration of MV
Baron, 2022 (12)	Brazil	NE	76	62.8 ± 17.4	38 (50.0%)	NMES	Frequency: 25 min, once a day, six times a week Intensity:100 Hz, pulse width of 500 μs	NE	73	63.7 ± 18.2	42 (57.5%)	Usual care	Mortality ICU LOS Duration of MV
Campos, 2022 (18)	Brazil	NE	34	42.5 ± 14.9	10 (30%)	NMES	Frequency: once a day for 60 min, five days a week Intensity:80 Hz, pulse width of 400 μs	NE	40	46.7 ± 17.9	14 (35%)	Early mobilization	Hospital LOS ICU LOS Duration of MV
Cerqueira, 2018 (19)	Brazil	NE	26	41.80 (13.17)	18 (69.2%)	NMES	Frequency: twice a day, (2 × 60 min) Intensity:50-Hz, pulse width of 400 μs	NE	33	42.21 (14.36)	23 (69.7)	Usual care	ICU LOS MRC
Chen, 2019 (3)	China	20.8 ± 7.4	16	77.7 ± 14.3	8 (50.0%)	EMS	Frequency: two 30-min per day, 5 d/wk. for 2 wk. Intensity: 50 Hz, pulse width of 400 μs	20.3 ± 6.3	17	73.8 ± 17.8	9 (52.9%)	Usual care	Mortality Duration of MV ICU LOS MRC
Chen A, 2019 (20)	China	19.81 ± 4.42	27	62.40 ± 13.60	14 (51.8%)	NMES	Frequency: 30 min twice daily until the patient was transferred from the ICU Intensity:30 to 40 Hz	19.03 ± 4.23	29	59.83 ± 11.75	17	Usual care	MRC ICU LOS Duration of MV Hospital LOS
Dall’ Acqua, 2017 (21)	Brazil	26 (5)	11	56 (13)	4 (36.3%)	NMES	Frequency: 30 min. One minute was added every 2 days of administration. Intensity: 50 Hz, pulse width of 300 μs	29 (7)	14	61 (15)	5 (35.7%)	Usual care	ICU LOS Duration of MV Mortality
Fischer, 2016 (17)	Austria	NE	27	63.3 (15.5)	18 (66.7%)	NMES	Frequency: twice a day (2 × 30 min) Intensity:66 Hz, pulse width of 400 μs	NE	27	69.7 (13.1)	20	Sham	Mortality MRC ICU LOS Duration of MV
Fossat, 2018 (22)	France	NE	158	65 (13)	103 (65%)	EMS	Frequency: 50 min Intensity: a 4-channel electrical simulator	NE	154	66 (15)	98 (64%)	Usual care	MRC Mortality
Gerovasili1, 2009 (23)	Greece	19 ± 3	13	59 ± 23	7 (53.8%)	NMES	Frequency: 55 min, the second to ninth day Intensity: 45 Hz, pulse width of 400 μs	18 ± 6	13	56 ± 19	5	Usual care	Duration of MV
Kho, 2015 (16)	USA	25 (8)	16	54 (16)	9 (56%)	NMES	Frequency: daily, 60 min Intensity: 50 Hz, pulse width of 400 μs	25 (6)	18	56 (18)	8 (50%)	Sham	Duration of MV ICU LOS Hospital LOS Mortality,
Liu, 2023 (24)	China	20.55 ± 6.40	40	58.13 ± 15.54	15 (37.5%)	NMES	Frequency: daily, sessions for 55 min Intensity:50 Hz, pulse duration 300 ms	19.78 ± 6.44	40	59.08 ± 16.02	17 (42.5%)	Usual care	Duration of MV MRC score ICU LOS Hospital LOS
Mahran, 2023 (25)	Egypt	12.28 ± 4.15	60	31 ± 10	14 (23.3%)	NMES	Frequency: daily, sessions for 55 min Intensity: 46 Hz	15.41 ± 7.30	58	31 ± 10	8 (13.8%)	Usual care	Mortality ICU LOS Duration of MV
McCaughey, 2019 (26)	Australia	NE	10	56.5 (18.50)	3 (30%)	FES	Frequency: 30 min, twice per day, days per week Intensity: 30 Hz, pulse width of 350 μs	NE	10	61.0 (17.25)	5 (50%)	Usual care	Mortality ICU LOS Duration of MV
Nakamura, 2019 (27)	Japan	22.8 (6.2)	21	76.6 (11.0)	14 (66.7%)	EMS	Frequency: once a day, 20 min Intensity: 20 Hz, pulse width of 250 μs	22.9 (3.9)	16	74.6 (13.1)	11 (68.8%)	Usual care	ICU LOS Duration of MV Mortality
Othman, 2023 (28)	Egypt	28.03 ± 4.42	30	40.30 ± 9.252	15 (50.0%)	NMES	Frequency: daily, sessions for 10 min Intensity: 50 Hz, pulse width of 400 μs	40.30 ± 9.252	30	42.43 ± 13.153	16 (53.3%)	Usual care	MRC scores ICU LOS Duration of MV ICU-AW
Patsaki, 2017 (29)	Greece	14 (9)	63	53 ± 15	19 (30%)	NMES	Frequency: daily for 55 min Intensity: 45 Hz, pulse width of 400 μs	17 (9)	65	53 ± 16	26 (40%)	Usual care	MRC Hospital LOS
Routsi, 2010 (30)	Greece	18 ± 4	68	61 ± 19	22 (32.3%)	EMS	Frequency: daily, 55 min Intensity: 45 Hz, pulse width of 400 μs	18 ± 5	72	58 ± 18	23	Usual care	Mortality MRC ICU LOS Duration of MV
Santos, 2020 (31)	Brazil	15.9 (3.2)	11	50.2 (12.8)	7 (66.66%)	NMES	Frequency: twice-daily Intensity: 45 Hz, pulse width of 400 μs	15.3 (3.7)	15	51.8 (12.8)	11 (73.33%)	Usual care	ICU LOS Mortality Duration of MV
Silva, 2019 (32)	Brazil	11 (8–13)	30	30 (27 to 33)	26 (13%)	NMES	Frequency: once a day for 25 min Intensity: 100 Hz, pulse width of 400 μs	11 (9–14)	30	33 (29 to 37)	26 (13%)	Usual care	Duration of MV ICU LOS Hospital LOS Mortality
Sumin, 2020 (33)	Russian	NE	18	61.5 (52.0;71.0)	12 (66.7%)	NMES	Frequency: daily, 90 min each Intensity: 45 Hz	NE	19	64.0 (60.0;68.0)	13 (68.4%)	Usual care	ICU LOS Hospital LOS
Vieira, 2023 (34)	Brazil	16.1 ± 4.6	20	34.7 ± 11.2	4 (20%)	NMES	Frequency: daily, sessions for 55 min Intensity: 50 Hz, pulse width of 400 μs	16.7 ± 4.5	20	36.5 ± 13.5	4 (20%)	Usual care	Mortality ICU LOS Duration of MV Hospital LOS
Waldauf, 2021 (35)	Czech Republic	22.1 ± 5.2	75	59.9 ± 15.1	22 (29.3%)	FES	Frequency: daily, 90 min Intensity: 40 Hz, pulse width of 250 μs	22.2 ± 7.7	75	62.3 ± 15.4	18	Usual care	Mortality MRC

NMES, Neuromuscular electrical stimulation; EMS, electrical muscle stimulation; FES, Functional electrical stimulation; APACHE II, Acute physiology and chronic health evaluation II; MV, Mechanical ventilation; MRC, Medical research council; ICU, Intensive care unit; LOS, Length of stay. Data are provided as means (standard deviation), means (95% confidence interval) or medians (interquartile range). NE, not estimable.

**Table 2:** Sensitivity analysis (omitting a signal RCT).

Selected study omitted		Hospital LOS (95%CI)	ICU LOS (95%CI)
Nakamura, 2019 (27)	EMS	/	−11.0 (−21.12 to −0.88)
Mahran, 2023 (25)	Total	/	−1.02 (−1.96 to −0.08)
Sumin, 2020 (33)	NMES	−2.36 (−3.85 to −0.88)	/
	Total	−2.38 (−3.81 to −0.94)	/

NMES, neuromuscular electrical stimulation; EMS, electrical muscle stimulation; ICU, intensive care unit; LOS, length of stay. Data are provided as mean differences (95% confidence interval); /, no statistical difference.

**Figure 2:**
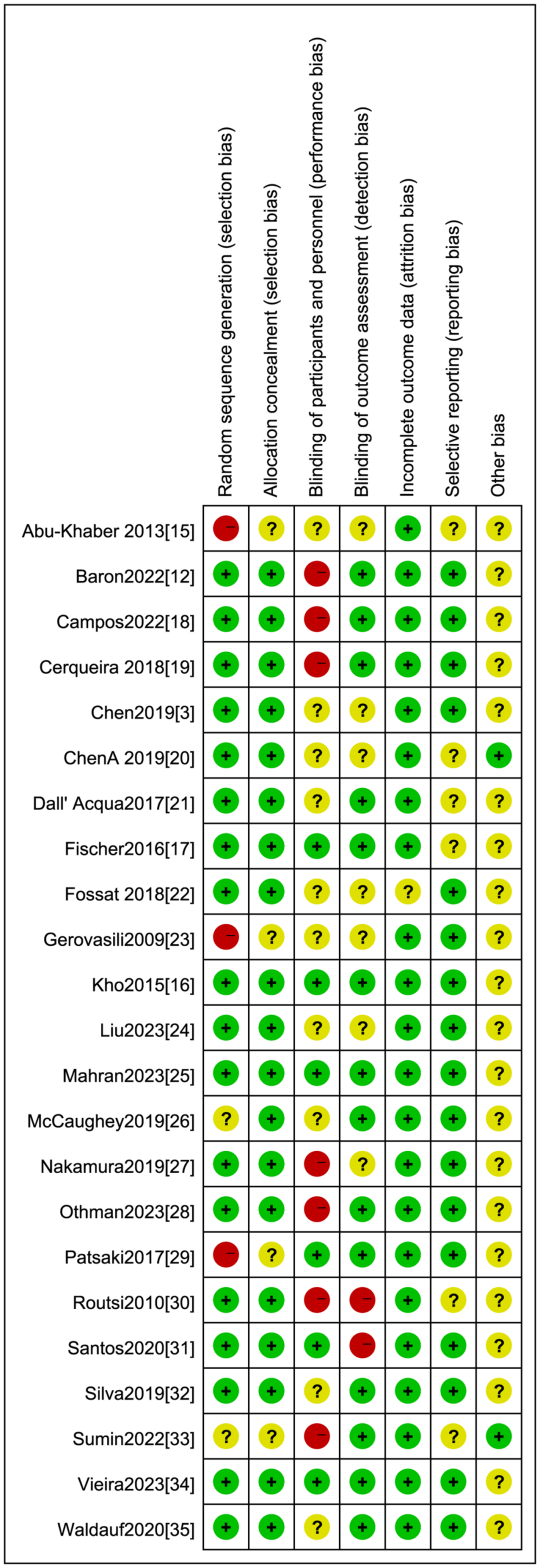
Risk of bias summary: Low risk of bias: Unclear: High risk of bias.

We appraised the included studies against seven domains for the risk of bias, which we categorized as ‘low’, ‘high’, or ‘unclear’. The results from these individual studies are summarized in Figure 2. We found that the method of randomized controlled allocation was flawed in three studies and was not expressly delineated in two studies. In six studies, the participants and personnel were not blinded, and in two studies, the outcome measures were not blinded.

### Outcome results

#### Mortality and duration of MV

**Figure 3:**
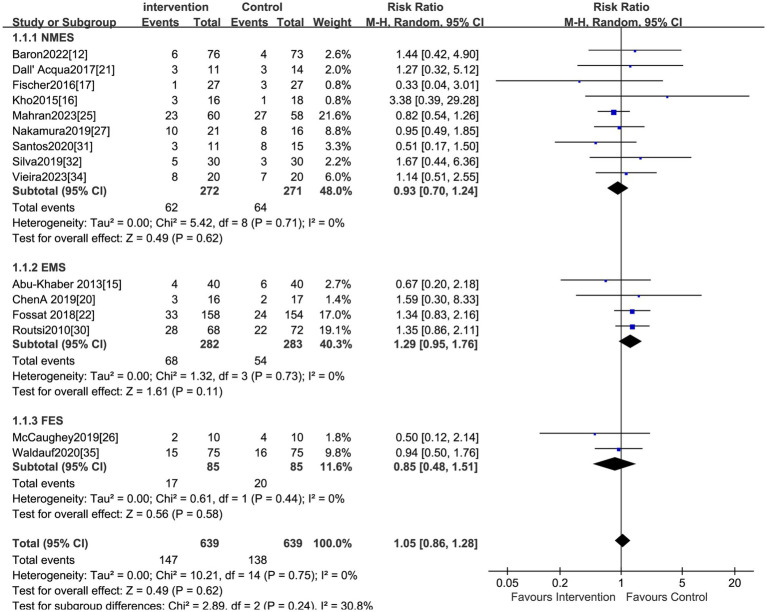
Forest plot of duration of mortality.

**Figure 4:**
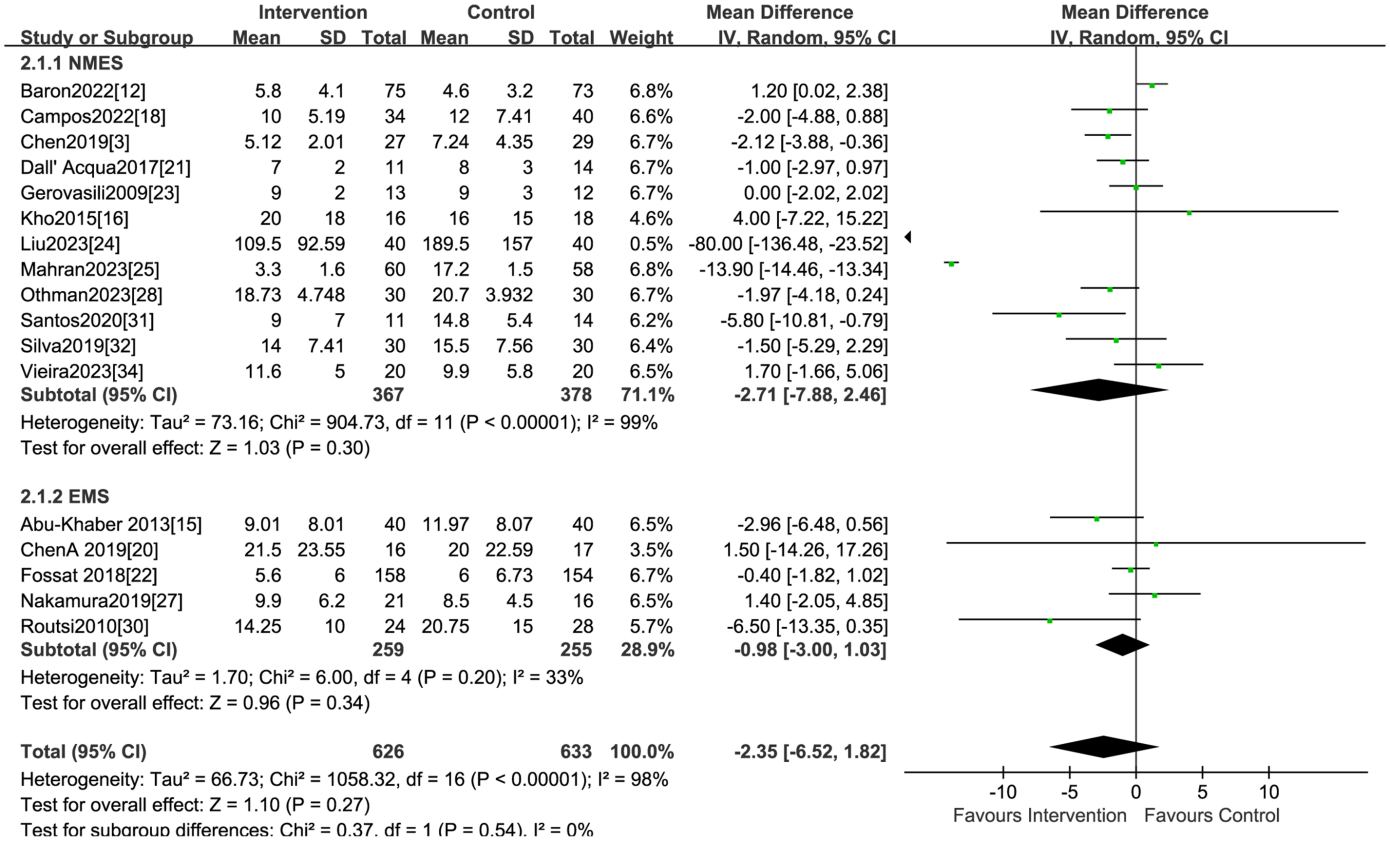
Forest plot of duration of MV.

The effect of electrical stimulation on mortality was reported in fifteen studies, including 1,278 patients. The results showed no difference in mortality in the electrical stimulation group compared to the control group (RR = 1.05,95% CI 0.86 to 1.28, *p* = 0.62) (Figure 3) with acceptable heterogeneity (I^2^ = 0%). Seventeen studies reported the effect of electrical stimulation on the duration of MV. The comparison between the electrical stimulation group and the control group showed no significant difference in the MV time (MD = −2.35, 95% CI −6.52 to 1.82, *p* = 0.27, I^2^ = 98%) (Figure 4). The subgroup and sensitivity analysis for EMS, FES, and NMES outcomes showed no differences in the mortality and the duration of MV.

### ICU LOS

**Figure 5:**
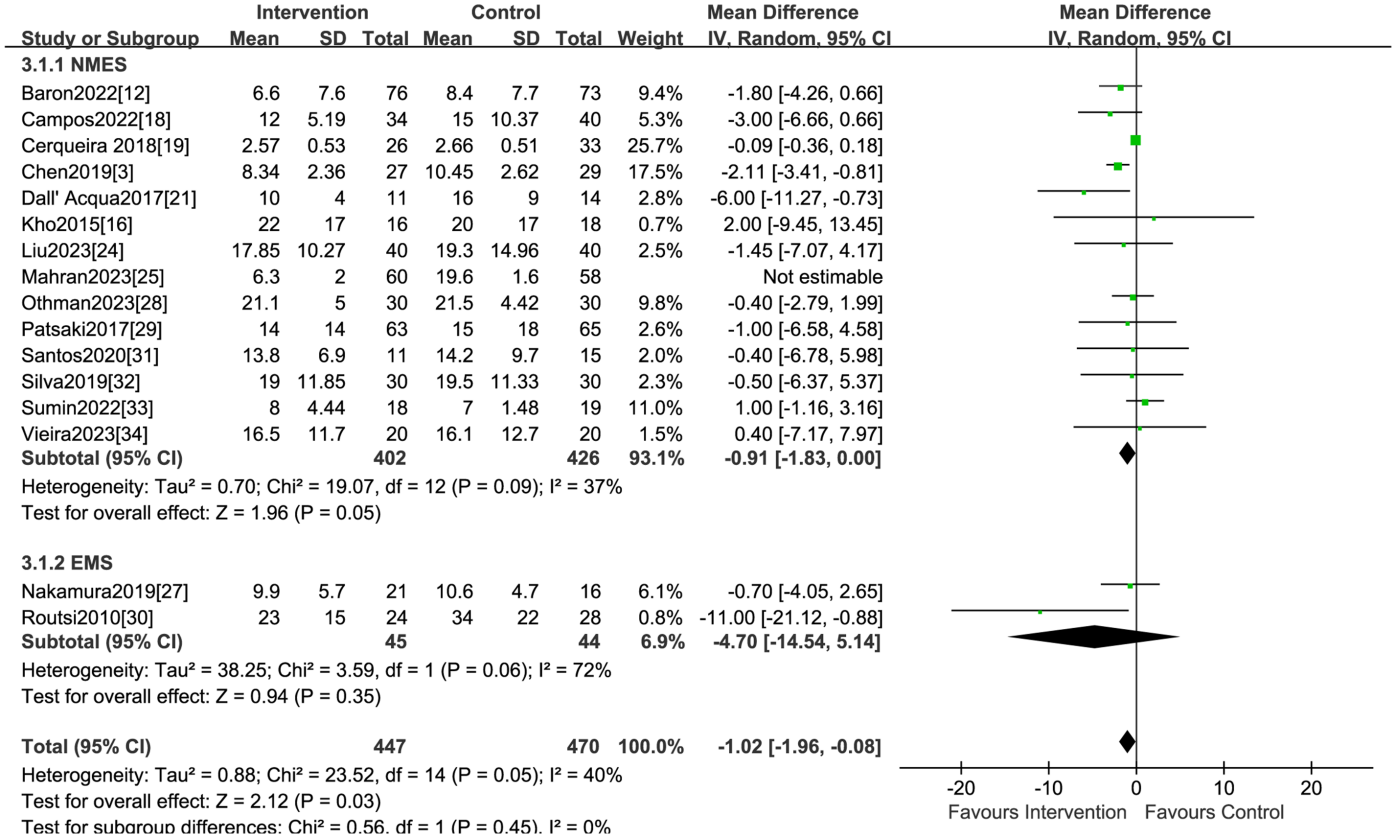
Forest plot of ICU LOS.

Sixteen studies reported the effect of electrical stimulation on ICU LOS. The results showed that there was no statistical difference in the electrical stimulation group compared with the control group (MD = −2.41, 95% CI −6.23 to 1.42, *p* = 0.22, I^2^ = 99%) (Figure 5). The subgroup analysis of EMS showed no differences in ICU LOS, but the sensitivity analysis reported the EMS could decrease the ICU LOS after excluding the study by Nakamura et al. (27) (MD = −11.0, 95% CI −21.12 to −0.88, *p* = 0.03). Nakamura et al. (27) primarily focused on patients with low APACHE II scores, and the ICU LOS was shorter than in other ICUs, which may be the main source of heterogeneity. The sensitivity analysis showed that the electrical stimulation could decrease the ICU LOS compared with the control group after excluding the study by Mahran et al. (25) (MD = −1.02, 95% CI −1.96 to −0.08, *p* = 0.03) (Table 2). Mahran et al. (25) focused on adult patients who were admitted to the ICU and required MV on the first day of admission, and aimed to evaluate the short-term outcomes of electrical stimulation in critically ill patients.

### Hospital LOS

**Figure 6:**
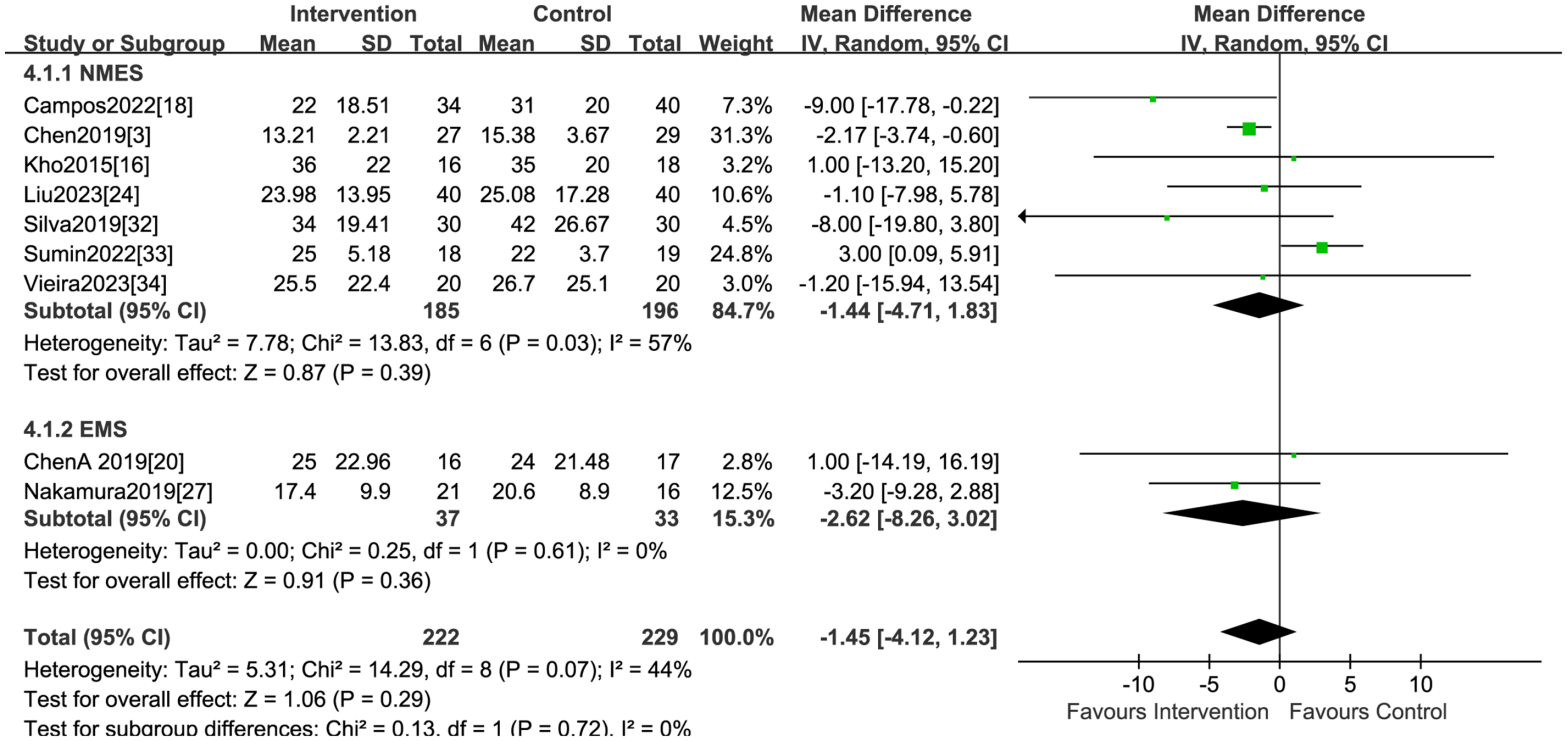
Forest plot of hospital LOS.

A total of nine studies, encompassing 451 patients, explored the impact of electrical stimulation. Statistical difference was not observed in hospital LOS when compared to the control group (MD = −1.45, 95% CI −4.12 to 1.23, *p* = 0.29, I^2^ = 44%) (Figure 6). The subgroup analysis of EMS showed no differences in hospital LOS, but the sensitivity analysis was conducted and identified the study by Sumin et al. (33) as the primary source of heterogeneity. Excluding this study, the results indicated the electrical stimulation (MD = −2.38, 95% CI −3.81 to −0.94, *p* = 0.001) and the subgroup of NMES (MD = −2.36, 95% CI −3.85 to −0.88, *p* = 0.002) led to a reduction in hospital LOS in comparison to the control group (Table 2). The research by Sumin et al. (33), which assessed the efficacy of NMES during the initial rehabilitation phase for patients experiencing postoperative complications following cardiovascular surgery, did not blind the assessors of muscle strength, which is presumed to be a significant source of heterogeneity.

### MRC scores

**Figure 7:**
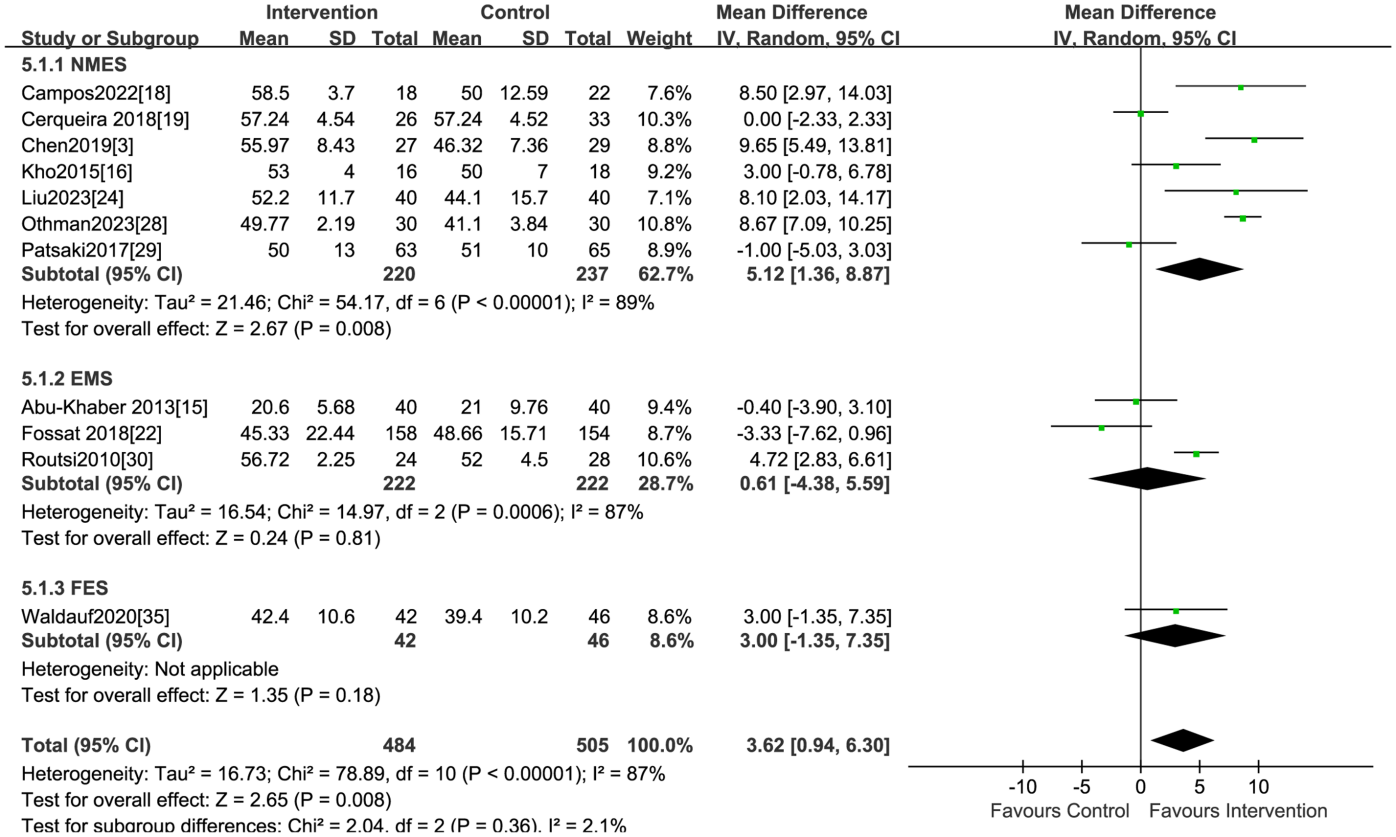
Forest plot of MRC scores.

Eleven studies, including 989 patients, reported the effect of electrical stimulation on MRC scores. The results showed that the MRC scores were significantly improved in the electrical stimulation group compared with the control group (MD = 3.62, 95% CI 0.94 to 6.30, *p* = 0.008, I^2^ = 87%) (Figure 7). The subgroup analysis of NEMS showed differences on MRC scores (MD = 5.12, 95% CI 1.36 to 8.87, *p* = 0.008, I^2^ = 89%) (Figure 7).

### Publication bias

Studies reporting on hospital LOS were less than ten, so we did not visualize the results using funnel plots. The funnel plots did not reveal obvious asymmetry for analyses on morality, duration of MV, MRC scores and the ICU LOS. Consistently, the Egger test suggested a lack of publication bias for morality (*p* = 0.25), duration of MV (*p* = 0.17), and ICU LOS (*p* = 0.15). Based on this analysis, the studies encompassed provided a broad coverage and produced statistically robust outcomes.

## Discussion

The objective of this review was to compile recent research on the effectiveness of electrical stimulation in boosting muscle strength and influencing various clinical outcomes in critically ill adults. We found that electrical stimulation significantly improves MRC scores, and there were no statistically significant differences in any other outcomes. Subgroup analysis suggests that NMES can effectively improve patients’ MRC scores. However, sensitivity analyses showed that electrical stimulation could reduce ICU LOS and hospital LOS. In addition, EMS effectively shortened the ICU LOS and NMES decreased the hospital LOS in sensitivity analyses.

Electrical stimulation methods, including NMES, EMS, and FES, utilize electrical currents to stimulate muscles or nerves, while these terms can be often utilized interchangeably, each has its own subtle differentiating uniqueness. NMES aims to restore voluntary movement by activating neurons that have lost their autonomous motor function, triggering skeletal muscle contractions. It also promotes neuromuscular and systemic blood flow, shielding neurons and muscle fibers from the negative effects of tissue hypoxia (36–39). By stimulating the peripheral nervous system, NMES can elicit various responses in the central nervous system, leading to neural adaptations (19–21). EMS, in comparison, focuses on maintaining or improving muscle tone and strength, particularly during periods of reduced physical activity, such as extended bed rest or critical illness (15, 27). It works by applying a series of electrical stimuli directly to skeletal muscles, inducing contractions and aiding in the restoration of muscle strength in critically ill patients. FES, meanwhile, employs carefully designed programs with low-frequency pulsed currents at specific intensities to stimulate and promote the recovery of impaired muscle functions (17). This targeted approach can be applied to individual or multiple muscle groups, with the goal of restoring function to the affected muscles.

Electrical stimulation has been applied to ICU patients as a safe, reliable, and effective way to accelerate their recovery (35). Compared to active training, electrical stimulation does not require patients’ participation and can be used in the very early stages of patient admission. Current evidence suggests that electrical stimulation is effective in preventing muscle atrophy in ICU patients, but there is no consistent conclusion on the effects of electrical stimulation on enhancing the motor function and reducing the duration of MV in ICU patients, and further studies are needed. Electrical stimulation aids in the promotion of muscle protein synthesis (40) and enhances muscle microcirculation in various acute conditions (38). As such, it has been adopted in the ICU as a method to counteract or reduce muscle wastage (41). The process involves triggering muscle contractions by sending electrical pulses through surface electrodes. This means there’s no need for the patient’s active participation, making it especially beneficial for those under continuous IV sedation or in early phases of acute diseases marked by delirium or significant unconsciousness. Recognizing this is crucial since muscle deterioration begins quickly, and the most substantial decline in muscle mass and function is seen within the first two weeks of ICU admission (42).

Previous meta-analyses of electrical stimulation have drawn various conclusions (11). The inclusion criteria for these previous meta-analyses differed from this current study regarding the types of interventions included (NMES, FES, and EMS compared to usual care only). Burke et al. (43) showed in a meta-analysis that, despite some conflicting individual study results, NMES in the ICU was significantly superior in preserving muscle strength, similar to our findings. In congruent with our finding, Lin et al. (44) reported that early implementation of NMES in ICU patients could prevent ICU-AW and improve their quality of life by enhancing their muscle strength and shortening ICU LOS. Furthermore, Zayed et al., in their 2020 study (2), reported that NMES did not reveal substantial variances in overall muscle strength and the length of stay in the hospital when compared to standard care in critically ill patients. This differs from our results, which indicates distinctions in hospital LOS. Cheng et al. (8) showed that NMES in the lower extremities could effectively shorten the duration of MV but had no significant advantages in increasing MRC scores, reducing ICU mortality, and shortening ICU LOS.

Our meta-analysis focused specifically on the effect of electrical stimulation within the ICU and included several recently published studies, and the results highlight a significant decreased in hospital LOS and improved MRC scores. Meanwhile, the subgroup of NMES showed differences in the outcomes. However, the sensitivity analysis demonstrated that EMS could shortened the ICU LOS and NMES decreased the hospital LOS.

### Strengths and limitations

The primary strength of this study is its inclusion of numerous RCTs, along with the use of subgroup and sensitivity analyses to minimize biases, thereby ensuring the reliability of the data and the stability of the results. By strictly adhering to the guidelines of the Cochrane Handbook, the research achieves a high degree of standardization and scientific rigor. The findings indicate that electrical stimulation can significantly improve the MRC scores in ICU patients, offering a practical therapeutic foundation for clinical application and potentially benefiting the rehabilitation process of critically ill patients. However, it’s important to consider potential limitations and the overall quality of the included studies to fully assess the reliability of these findings. Nevertheless, there were some limitations to this study. The included literature is only published in English, which may lead to publication bias due to an incomplete literature search. In addition, the sample size of individual studies is small, which may affect the analysis results. Furthermore, some studies were not blinded in the implementation of electrical stimulation and measurement results, which may lead to some implementation and measurement bias. Finally, the wide age range of the adult ICU patients on mechanical ventilation included in this study introduces significant heterogeneity, which is a significant limitation of our work. Future studies with larger samples and longer follow-ups are needed to comprehensively explore the short and long-term effects of electrical stimulation on patient outcomes to understand the intervention’s effects further.

## Conclusion

To conclude, the application of electrical stimulation in the ICU demonstrates a notable improve in MRC scores. In critical care, NMES is crucial for preventing muscle atrophy and improving recovery outcomes. By using NMES, muscles can be activated and maintained even when patients are unable to exercise actively. Nevertheless, given the constraints posed by the quality and sample size of the studies included, the broader impact should be substantiated through additional high-quality, large-scale, multi-center investigations.

## Data availability statement

The original contributions presented in the study are included in the article/supplementary material, further inquiries can be directed to the corresponding author.

## Author contributions

LL: Writing – review & editing, Writing – original draft, Supervision, Formal analysis, Conceptualization. FL: Writing – review & editing, Resources, Funding acquisition. XZ: Writing – original draft, Methodology, Investigation. YS: Writing – original draft, Software, Data curation. SL: Writing – original draft, Project administration, Methodology. HY: Writing – original draft, Supervision, Investigation.
